# Anxiety and Depressive Symptoms Correlated to Patient-Reported Outcome Measures after Rotator Cuff Repair: A Prospective Study in the Perioperative Period

**DOI:** 10.3390/jcm12082999

**Published:** 2023-04-20

**Authors:** Umile Giuseppe Longo, Sergio De Salvatore, Ilaria Piergentili, Nicolò Panattoni, Anna Marchetti, Maria Grazia De Marinis, Vincenzo Denaro

**Affiliations:** 1 Research Unit of Orthopaedic and Trauma Surgery, Fondazione Policlinico Universitario Campus Bio-Medico, Via Alvaro del Portillo, 200, 00128 Rome, Italy; ilaria.piergentili94@gmail.com (I.P.); denaro@policlinicocampus.it (V.D.); 2 Research Unit of Orthopaedic and Trauma Surgery, Department of Medicine and Surgery, Università Campus Bio-Medico di Roma, Via Alvaro del Portillo, 21, 00128 Rome, Italy; s.desalvatore@unicampus.it; 3 Department of Biomedicine and Prevention, University of Rome Tor Vergata, Via Montpellier 1, 00133 Rome, Italy; nicolo.panattoni@alumni.uniroma2.eu; 4 Research Unit Nursing Science, Campus Bio-Medico University, Via Alvaro del Portillo 21, 00128 Rome, Italy; a.marchetti@unicampus.it (A.M.); m.demarinis@unicampus.it (M.G.D.M.)

**Keywords:** hospital anxiety and depression scale, PROMs, anxiety, depression, rotator cuff tear, rotator cuff repair

## Abstract

Anxiety and depressive symptoms adversely affect surgical outcomes in patients with rotator cuff tear (RCT) undergoing surgical repair. Patients without a diagnosis of mood disorders, such as anxiety and depression, before rotator cuff repair (RCR) can be considered an optimal candidate for surgery. The objective of this prospective observational study was to evaluate the relationship between anxiety and depressive symptoms, using the Hospital Anxiety and Depression Scale (HADS) as an assessment tool, and patient-reported outcome measures in RCT after repair surgery. This study included patients with RCT undergoing arthroscopic rotator cuff repair (RCR). Forty-three patients were included who had completed the HADS, Constant Murley Score (CMS), and Short Form Health Survey 36 (SF-36) questionnaires before surgery and in the postoperative follow-up, at 1 month, 3 months, and 6 months. The Friedman test showed that there were statistically significant changes in the different times point for HADS (*p* < 0.001), anxiety subscale of HADS, i.e., HADS-A (*p* < 0.001), depression subscale of HADS, i.e., HADS-D (*p* < 0.001), CMS (*p* < 0.001), and SF-36 (*p* < 0.001). The average scores of HADS, HADS-A, and HADS-D improved at each follow-up, showing improvement in discomfort. From the third month after surgery, there was an improvement in anxiety and depression disorders related to improved quality of life, functionality, and pain perception. The trend remained stable until the sixth month of follow up. This study shows that anxiety and depressive symptoms in RCT patients are significantly reduced after RCR with subsequent important improvements in terms of functionality, ability to carry out activities of daily living, perceived pain, and quality of life.

## 1. Introduction

Anxiety and depressive symptoms adversely affect surgical outcomes in patients with rotator cuff tear (RCT) undergoing surgical repair [1,2,3,4,5,6]. The most common outcomes assessed in the RCT literature focus on pain and disability improvements after surgery [2]. A few studies assessed the preoperative and postoperative anxiety and depressive symptoms in RCT patients. In patients with shoulder diseases, anxiety and depressive symptoms are usually secondary to the pain and functional disability [4]. Frequently, functional limitation, chronic pain, insomnia, and other conditions related to RCT lead to anxiety and depression symptoms [4]. Furthermore, possessing mental disorders, such as anxiety and depression, in RCT patients and candidates for surgical repair significantly impacts the economic burden of care, which justifies necessary attention [7].

RCT is a highly prevalent condition among shoulder disorders, causing pain, functional impairment and strength deficit. Scientific evidence shows that approximately 65% of repair surgeries of RCTs are performed on patients who are aged <65 years, thus, predominantly affecting the working population. The alternative, conservative treatment, may predispose patients to continued irreversible tissue degeneration over time [8]. In addition to physical symptoms, rotator cuff tears can also have a significant psychological impact on patients. The pain and limited mobility associated with this injury can lead to frustration, fear, and uncertainty about the future. [6,9,10,11]. Rotator cuff repair (RCR) is the treatment of choice for chronic and symptomatic full-thickness RCT [12]. The surgical repair of the rotator cuff is an elective surgery, so proper preoperative preparation of the patient is essential, through careful evaluation, education, and discussion of the postoperative recovery period [13]. Psychosocial factors are crucial both in the preoperative and postoperative periods due to the significant impact on PROMs and postoperative recovery in RCR patients [6]. A structured and systematic approach to these factors, in combination with surgery, is necessary to improve recovery. With this in mind, the integration of a multidisciplinary team is necessary to assess the pathophysiological and psychosocial aspects completely [14]. In general, patients show a noticeable reduction of pain and improvement of functional capacity of the shoulder after surgical treatment [9,15]. Patients without a diagnosis of mood disorders, as anxiety and depression, before RCR can be considered optimal candidates for surgery [16,17]. Lau and colleagues [3] reported a significant correlation between post-surgical functional improvement and relief in anxiety and depression status.

Patient Reported Outcome Measures (PROMs) are considered subjective parameters commonly used in orthopaedic studies to assess health status and symptom evolution after surgery [18,19]. Furthermore, PROMs are increasingly being used to personalize a clinical-therapeutic path as well as in health policy decisions. There are several types of PROMs used in orthopedic surgery, including generic, disease-specific, and joint-specific measures. PROMs related to RCT include perceptions and opinions about symptoms, functionality, health-related quality of life, and satisfaction [18,20]. Despite the benefits of using PROMs, there are also challenges associated with their use. One challenge is selecting the appropriate PROM for a given condition or treatment. Another challenge is ensuring the validity and reliability of the data collected through PROMs [21]. Patients’ perspectives, experiences, and perceptions of their state of health have become essential in decision-making procedures and in evaluating the effectiveness of treatment. A preoperative anxiety and depression assessment of patients who are candidates for surgery could improve surgical outcomes and ensure support, education, and personalized treatment [22]. As the use of PROMs in orthopedic surgery continues to grow, research is needed to better understand their validity and usefulness. One topic to study is the PROMs’ treatment outcomes, including psychosocial factors and patient satisfaction. Another topic to study is the validation of existing PROMs across different patient populations and settings. Finally, studies are needed to explore the impact of PROMs on clinical decision-making and patient outcomes, as well as the cost-effectiveness of using PROMs in routine clinical practice [23].

While most studies on RCT surgery focus on pain and disability improvements after the procedure, a few have examined the impact of anxiety and depression on surgical outcomes [9,24]. Anxiety and depressive symptoms can significantly affect the success of RCT surgery, making it even more critical to address these issues before and after the procedure.

Anxiety and depression in RCT patients are often linked to the pain and functional limitations caused by the condition. Chronic pain, insomnia, and other symptoms related to RCT can lead to anxiety and depression, which only exacerbates these problems. This can become a vicious cycle that can be difficult to break without proper care and attention.

Mental disorders such as anxiety and depression can also significantly impact the economic burden of care of RCT patients and candidates for surgical repair. This is why it is essential to give mental health issues the attention they deserve when treating RCT patients.

Some authors have explored perioperative PROM changes [18,25,26]; however, our study differs from previous work in the assessment tools used, which specifically assess pain, function, strength, and disability in activities of daily living. The aim of the present study is to assess the relationship between anxiety and depressive symptomsand PROMs in RCT patients after surgery, by evaluating the correlation between Hospital Anxiety and Depression Scale (HADS) scores and Constant Murley Scores (CSM) and Short Form Health Survey 36 (SF-36) scores.

## 2. Materials and Methods

The Strengthening the Reporting of Observational Studies in Epidemiology (STROBE) guidelines guided the preparation of this document to assure the methodological quality of this prospective observational study.

From February 2019 to February 2020, 101 patients who underwent arthroscopic RCR were recruited from the Department of Orthopaedic and Trauma Surgery of the Campus Bio-Medico University Hospital in Rome, Italy. The study was conducted according to the guidelines of the Declaration of Helsinki and approved by the Institutional Review Board of Campus Bio-Medico University of Rome (COSMO study, Protocol number: 78/18 OSS ComEt CBM, 16/10/18).

Two orthopaedic surgeons specializing in shoulder arthroscopy performed the clinical examination and assessed the preoperative Magnetic Resonance Imaging (MRI). Only patients with Goutallier grade 2 and Patte stage 2 lesions were included in our study [16,27]. These classifications are based on fatty infiltration of the rotator cuff musculature (Goutallier classification) [16] and the amount of supraspinatus tendon retraction applied on sequences in the frontal plane (Patte classification) [23]. All patients received conservative treatments (physical therapy and corticosteroid injections). The same senior surgeon performed all the procedures. Patients not undergoing surgery or with other types of shoulder pathologies were excluded. All the patients included completed a standardized rehabilitation protocol [28]. The arm was supported with an abduction sling pillow for the first four weeks, and pendulum exercises, table slide, and active elbow extension and flexion were permitted. Exercises with small circular pendulums were carried out. In the table slide exercise, the patient advances the chest towards the table while sliding the hand of the operated shoulder forward on a surface. The patients began therapy four weeks after surgery, working with the therapist 1–3 times per week and at home on the other days. From week 5 to week 8, the patients performed passive forward elevation, passive external rotation, and, beginning in week 5, active assisted range of motion (ROM) to tolerance. Patients advanced to active ROM tolerance from week 8 to week 10. After week 10, patients began concentric and eccentric workout strengthening for the deltoid, scapular stabilizers, and rotator cuff.

All of these patients completed HADS, CMS, and SF-36 questionnaires before surgery. Patients were asked to complete the same questionnaires before surgery and at 1 month, 3 months, and 6 months after surgery. Patients were included in the study if they had completed up to 6 months of post-surgery follow-up. Demographic and surgical characteristics were recorded prospectively.

HADS is a reliable rating scale for detecting perceived anxiety and depression symptoms levels in various patient clinical specialities [29,30,31]. It is also used to monitor these psychological symptoms over time [31]. The questionnaire consists of 14 items divided into two 7-element scales: a scale for evaluating anxiety (HADS-A) and one for evaluating depression (HADS-D). By summing scores for each item, the overall score ranges from 0 to 21. The severity of depression or anxiety is classified as 0 to 7, normal; 8 to 10, mild case; 11 to 15, moderate case; and 16 to 21, severe case [30]. Hence, a lower score represents a better condition.

The CMS is a validated measure of a patient’s shoulder pain, function, and capacity to carry out daily activities [32]. It was developed to measure the functionality after the treatment of a shoulder injury. The score can range from 0 to 100. A higher score represents a better condition.

The SF-36 is a 36-item questionnaire that evaluates eight health status parameters and quality of life: physical functioning, role limitations due to emotional or physical problems, social functioning, mental health, physical pain, vitality, and general health perceptions [33]. The score ranges from 0 to 100. A higher score represents a better condition.

### Statistical Analysis

Data normality was assessed using Shapiro-Wilk tests of normality. Since data did not respect the normality distribution, baseline and postoperative follow-up scores were compared using the Friedman and Wilcoxon Signed Ranks Tests with Bonferroni correction. Tests for correlation between anxiety and depressive symptoms and the Constant scores and SF-36 questionnaire scores at the last follow-up were performed using the Spearman Rank Correlation Test (Spearman’s Rho). The statistical level of significance was 0.05.

All data were analysed using IBM SPSS Statistics for Windows, Version 26.0. (IBM Corp: Armonk, NY, USA) and Statistical Analysis System (SAS) OnDemand for Academics.

An a priori power analysis, similar to that conducted in the literature [34], was performed for a Cohen’s d effect size of 0.4 in the HADS for depression from baseline to 6 months. For an alpha level of 0.05 and a power of 80%, a sample size of 41 patients was estimated to be required. All enrolled patients had an RCT with Goutallier grade 2 and Patte stage 2.

## 3. Results

Only 43 patients (21 females and 22 males), with mean age 63.3 ± 11.1 years, completed all questionnaires up to the 6-month follow-up and were included in this study.

The Friedman test showed that there were statistically significant changes at the different time points for HADS (*p* < 0.001), HADS-A (*p* < 0.001), HADS-D (*p* < 0.001), CMS (*p* < 0.001) and SF-36 (*p* < 0.001) (Table 1).

Statistically significant differences in most scores were found between preoperative and postoperative time points using pairwise comparisons (Wilcoxon test with Bonferroni correction) (Table 2). Exceptions were in SF-36 scores: between preoperative and 1-month follow-up, between preoperative and 3 months follow-up (*p* = 0.796), between preoperative and 6 months follow-up (*p* = 0.675), and between 3 months to 6 months postoperative follow-ups (Table 2).

**Figure 1:**
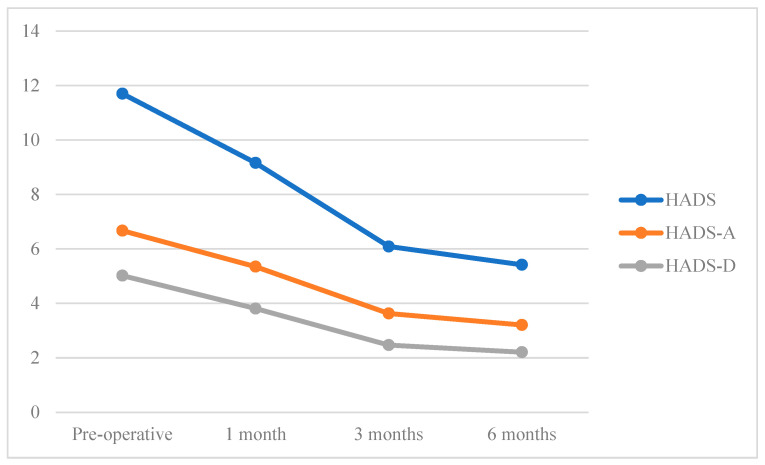
HADS, HADS-A and HADS-D follow-up.

The average scores of HADS, HADS-A and HADS-D decreased at each follow up (Figure 1).

**Figure 2:**
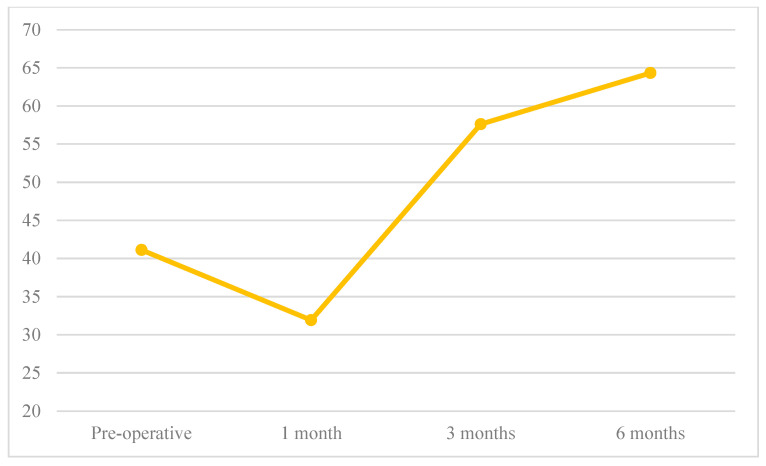
Constant Score follow-up.

**Figure 3:**
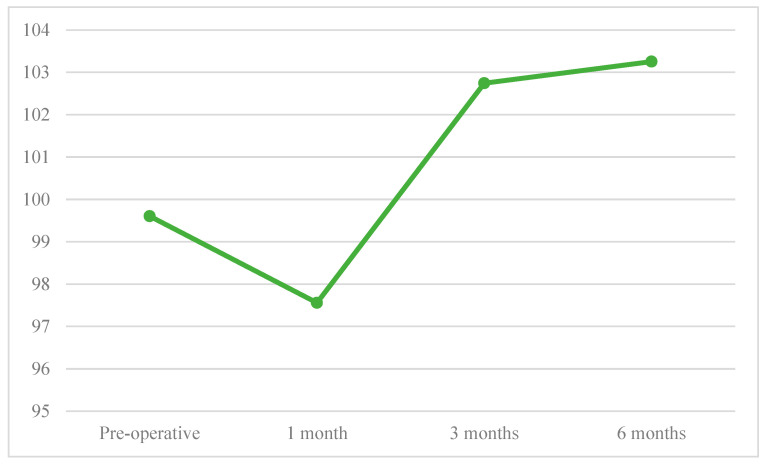
SF-36 follow-up.

The mean values of CMS and SF-36 score decreased between preoperative to 1-month follow-up and increased at 3-and 6-months postoperative follow-ups (Figure 2 and Figure 3).

Therefore, a worsening was observed in the short term (within the first month) but improved from the third month onwards. However, the difference between the preoperative and 1-month follow-up scores was not statistically significant.

Statistically significant correlations were found between HADS, HADS-A, and HADS-D and CMS and SF-36 in the preoperative and postoperative follow-ups (Table 3), except at a 1-month postoperative time point for CMS. As HADS, HADS-A, and HADS-D increased, CMS and SF-36 decreased.

## 4. Discussion

The present study assessed the relationship between anxiety and depressive symptoms and PROMs in RCT patients after surgical repair, with some differences in results compared to previous studies [18,25,26]. From the third month after surgery, there was an improvement in anxiety and depression disorders related to the improved quality of life, functionality, and pain perception. The trend remained stable until the sixth month of follow up.

The results of the present study are similar to those in other studies on the topic published in the literature, but with some differences, which reflects the originality of, and confers clinical relevance to the present study. In our study we correlated the HADS rating scale with CMS, which allowed us to specifically assess pain, function, strength and disability in activities of daily living. Our results show not only an overall improvement in clinical outcomes after RCR in terms of shoulder function and movement, but also a substantial improvement in the ability to perform activities of daily living. This further strengthens the evidence for the improvement in terms of quality of life.

Cho and colleagues [9] analysed the correlation between anxiety, depression, and PROMs up to 12 months of follow up in a sample of 47 patients. The authors reported that arthroscopic rotator cuff repair had been shown to positively impact patients’ physical and mental Health-Related Quality of Life (HRQOL), as measured by the SF-36. Factors that played a role in postoperative HRQOL include demographics such as age, sex, medical comorbidities such as Diabetes Mellitus (DM), and level of sports activity. Interestingly, the size of the rotator cuff tear, fatty infiltration of the rotator cuff muscles, symptom duration, and repair integrity were not significant predictors of HRQOL. Surgeons should consider these clinical factors when planning for rotator cuff repair surgery. Targeted protocols for surgery and rehabilitation, as well as prognosis evaluation, could be developed as a result.

As reported in the present study and in the recent study by Thorpe et al., improvements in mood disorders occurred from the third month. Thorpe et al. [18] demonstrated the negative influence of anxiety and depression disorders on surgical outcomes. The authors highlighted that low preoperative psychological health scores correlate with poor surgical outcomes up to one year following surgery. Thorpe and colleagues suggested assessing the preoperative psychological status to consider corrective psychological treatments before surgery. In their study, the authors identified two groups. One group had poorer mental health before their shoulder surgery, which was linked to higher levels of pain and disability both before and up to a year after the surgery. This was found after considering factors such as gender, workers’ compensation claim, alcohol consumption, and confidence in the outcome of the surgery. However, both groups showed similar improvements in their ASES score over time. This study looked at both affective and cognitive mental health measures, a broader analysis than previous studies on shoulder surgery. Nevertheless, the results support recent findings that depression and catastrophising are linked to higher pain and disability in the shoulder and other musculoskeletal conditions.

Cho et al. [29] reported that pain persistence for more than three months after surgery was associated with anxiety and/or depression. In their study, the authors reported that the self-evaluation of patients prior to rotator cuff repair was negatively affected by their experience of depression and anxiety. Specifically, depression was found to strongly predict the patients’ perception of functional disability and their health-related quality of life (HRQOL). This indicates that a patient’s psychological state before the surgery may be crucial in determining the outcome of the surgery, making psychological assessment an essential aspect of preoperative care.

Similarly, Lau et al. [3] analysed the scores of PROMs related to anxiety and depressive symptoms in a study of 171 patients undergoing RCR. They stated that both patients with previous diagnoses of anxiety and depression and patients without psychological diseases reported improvements in PROMs after RCR. However, the former group reported lower results. These findings highlighted the importance of psychological assessment before surgery to obtain the best results possible after RCR.

The economic burden of RCRs affects the healthcare system and a significant growth trend is expected in the coming years [12]. Cronin et al. [5] examined the changes in healthcare costs before and after an RCR, specifically focussing on the predominance of anxiety and depressive symptoms in treated patients. Cronin reported that patients with anxiety or depression reported higher healthcare costs compared to healthy patients. This was likely due to opioid use and hospital readmission rate of these patients. Therefore, finding valid methods to assess the preoperative psychological state of patients could lead to the adoption of specific early treatment for those patients who show psychological distress, with a subsequent reduction in health care costs for the health system after surgery.

In agreement with the findings of this study, recent scientific literature suggests an adequate evaluation of mood disorders in the postoperative period. Park et al. [35], adopted the same assessment tool as the present study (HADS) to assess the influence of anxiety and depressive symptoms on RCR outcomes. Their study highlighted how anxiety and depressive symptoms negatively influenced the clinical results after RCR, specifically pain, function, and range of motion. Therefore, the authors concluded that an assessment of the preoperative psychological status was necessary for RCR surgery. Cho et al. [2] and Woollard et al. [31] reported similar conclusions, considering depression a strong predictor of poor health-related quality of life after surgery. The latter author stated that the predictive ability of psychosocial factors and shoulder impairments in determining successful outcomes following elective surgery for rotator cuff pathology has rarely been studied. However, by utilising information collected during the preoperative examination, accurate predictions can be made of the patient’s condition six months after surgery. While measures of shoulder impairment and rotator cuff damage were not strong predictors of patient-reported outcomes, high fear-avoidance scores, particularly on the work subscale, and the surgery performed on the dominant shoulder were found to be strong predictors. Fear-avoidance scores can be easily obtained from patients using PROMs, such as the Fear Avoidance Beliefs Questionnaire (FABQ), during clinic visits.

Preoperative patient depression is a significant factor for patient-reported outcomes in patients after rotator cuff surgery. A retrospective study by Johnson and colleagues shows that preoperative depression is an independent predictor of persistent postoperative pain [7]. In addition, their research shows that patients who experience depression and anxiety prior to arthroscopic RCR surgery tend to have lower postoperative scores for Upper Extremity Function and higher probability of postoperative complications. Their study also suggests that preoperative depression and anxiety can predict whether the pain will persist after the surgery. Although depression and anxiety should not stop a patient from having RCR surgery, additional treatments may be necessary to promote positive outcomes, lower complications, and decrease healthcare needs.

In light of the literature reviewed, it is evident that it is clinically relevant to implement evidence on the topic of PROMs and orthopaedic surgery. A cross-sectional study, aimed at identifying the prevalence of PROM use by orthopaedic surgeons, shows that few studies focus on RCT. Furthermore, the results testify to the low use of PROMs in clinical practice is caused by lack of knowledge, beliefs that collecting PROMs is too time-consuming and requires a costly overhaul to the structure of their clinical activity [23].

### Limitations

The first limitation of this study was the lack of a control group to compare HADS scores in a conservatively treated RCT cohort.

The second limitation was that patients with a specific diagnosis of anxiety or depression were not divided into a separate group. Our study aimed to specifically assess anxiety and depressive symptoms with pain, function, strength and disability in activities of daily living. This research suggests that improvements in anxiety and depressive symptoms can be considered as effects of stable improvements in surgical outcomes. As underlined, anxiety and depressive symptoms adversely affect surgical outcomes in RCR patients [1,2,3,4,5,6]. In patients with shoulder diseases, anxiety and depressive symptoms are usually secondary to pain and functional disability [4]. Specifically, functional limitation, chronic pain, insomnia, and other conditions related to RCT lead to anxiety and depression symptoms [4]. In the present study, participants completed HADS, CMS, and SF-36 questionnaires before surgery and then at 1 month, 3 months, and 6 months after surgery rotator cuff repair. 

The third limitation was the choice of follow-up time. The 6 month period we chose should not be considered a final follow-up, but was an adequate observation period that allowed us to draw the first significant results regarding anxiety and depression in the strictly perioperative period. Moreover, the duration of the RCR tear could affect the depression condition. The rate of work-related injuries was not reported in our database. Increasing the follow-up period to 2 years might be desirable in future studies.

Hence, the study authors suggest prudence in generalising these study results, although from the third month after surgery there was an improvement in anxiety and depression disorders related to the improved quality of life, functionality and pain perception.

## 5. Conclusions

The results of our study permitted us to assess the relationship between anxiety and depressive symptoms and PROMs in RCT patients after surgery, by evaluating the correlation between HADS scores and CSM and SF-36 scores.

This study shows that anxiety and depressive symptoms in RCT patients are significantly reduced in the RCR perioperative period with subsequent important improvements in terms of functionality, ability activities of daily living, perceived pain and quality of life. Despite the study limitations, it is essential to consider the clinical relevance of the results of this study. Anxiety and depressive symptoms appear to be correctable after RCR in RCT patients candidate for surgery. This entails a drastic improvement in surgical outcomes, such as the quality of life. Through the assessment and proper treatment of preoperative mental status, patients could manage their emotional distress and improve the recovery process.

Future research should focus on early screening and effective management of anxiety and depressive symptoms from the preoperative period in RCR-indicated patients to get better surgical outcomes for RCT patients. As research in this field continues to evolve, PROMs will play an increasingly important role in improving patient care and outcomes in orthopedic surgery.

## Figures and Tables

**Table 1:** Statistically significant changes for HADS, HADS-A, HADS-D, CMS, and SF-36.

	Time	N	Mean	SD	Minimum	Maximum	*p*-Value, Friedman Test
HADS	Pre-operative	43	11.7	8.7	0	32	<0.001 *
	1 month	43	9.2	7.4	0	29	
	3 months	43	6.1	6.8	0	27	
	6 months	43	5.4	6.9	0	27	
HADS-A	Pre-operative	43	6.7	4.6	0	16	<0.001 *
	1 month	43	5.4	3.8	0	14	
	3 months	43	3.6	3.4	0	13	
	6 months	43	3.2	3.5	0	13	
HADS-D	Pre-operative	43	5.0	4.5	0	18	<0.001 *
	1 month	43	3.8	4.3	0	17	
	3 months	43	2.5	3.7	0	16	
	6 months	43	2.2	3.7	0	16	
CONSTANT SCORE	Pre-operative	43	41.1	16.7	4	70	<0.001 *
	1 month	43	31.9	10.3	9.5	60	
	3 months	43	57.6	12.9	23.5	75	
	6 months	43	64.3	9.9	41.5	77	
SF-36	Pre-operative	43	99.6	8.5	85	116	<0.001 *
	1 month	43	97.6	7.7	79	111	
	3 months	43	102.7	7.5	80	116	
	6 months	43	103.3	6.9	80	116	

Note: (*) = statistically significant.

**Table 2:** Statistically significant differences between preoperative and postoperative time points.

Score	Pre-op vs. 1 Month	Pre-op vs. 3 Months	Pre-op vs. 6 Months	1 Month vs. 3 Months	1 Months vs. 6 Months	3 Months vs. 6 Months
HADS	0.012 *	<0.001 *	<0.001 *	0.001 *	<0.001 *	0.146
HADS-A	0.040 *	<0.001 *	<0.001 *	<0.001 *	<0.001 *	0.140
HADS-D	0.021 *	<0.001 *	<0.001 *	0.006 *	<0.001 *	0.268
CONSTANT SCORE	0.004 *	<0.001 *	<0.001 *	<0.001 *	<0.001 *	<0.001 *
SF-36	0.061	0.033 *	0.014 *	<0.001 *	<0.001 *	0.495

Note: (*) = statistically significant.

**Table 3:** Statistically significant correlations between HADS, HADS-A, HADS-D, CMS and SF-36. Each comparison is made between the score at the same follow-up.

		HADS Pre		HADS 1 mo		HADS 3 mo		HADS 6 mo
rho	Constant score Pre	−0.4	Constant score 1 mo	−0.2	Constant score 3 mo	−0.5	Constant score 6 mo	−0.4
*p*-value		0.008 *		0.152		0.002 *		0.007 *
rho	SF-36 Pre	−0.6	SF-36 1 mo	−0.5	SF-36 3 mo	−0.4	SF-36 6 mo	−0.6
*p*-value		<0.001 *		0.001 *		0.008*		<0.001 *
		HADS-A Pre		HADS-A 1 mo		HADS-A 3 mo		HADS-A 6 mo
rho	Constant score Pre	−0.3	Constant score 1 mo	−0.2	Constant score 3 mo	−0.4	Constant score 6 mo	−0.4
*p*-value		0.032 *		0.255		0.003 *		0.016 *
rho	SF-36 Pre	−0.5		−0.5	SF-36 3 mo	−0.4	SF-36 6 mo	−0.6
*p*-value		<0.001 *	SF-36 1 mo	0.001 *		0.006 *		<0.001 *
		HADS-D Pre		HADS-D 1 mo		HADS-D 3 mo		HADS-D 6 mo
rho	Constant score Pre	−0.5	Constant score 1 mo	−0.3	Constant score 3 mo	−0.5	Constant score 6 mo	−0.5
*p*-value		0.001 *		0.099		0.001 *		0.002 *
rho	SF-36 Pre	−0.6	SF-36 1 mo	−0.4	SF-36 3 mo	−0.5	SF-36 6 mo	−0.5
*p*-value		<0.001 *		0.014 *		0.001 *		<0.001 *

Note: (*) = statistically significant.

## Data Availability

The datasets used and/or analysed during the current study are available from the corresponding author on reasonable request.
